# Arabidopsis FIM5 decorates apical actin filaments and regulates their organization in the pollen tube

**DOI:** 10.1093/jxb/erw160

**Published:** 2016-04-25

**Authors:** Meng Zhang, Ruihui Zhang, Xiaolu Qu, Shanjin Huang

**Affiliations:** ^1^Key Laboratory of Plant Molecular Physiology, Institute of BotanyChinese Academy of Sciences, Beijing 100093China; ^2^Center for Plant Biology, School of Life Sciences, Tsinghua UniversityBeijing 100084, China; ^3^Tsinghua-Peking Joint Center for Life Sciences, Beijing 100084China; ^4^University of Chinese Academy of Sciences, Beijing 100049China; ^5^National Center for Plant Gene Research, Beijing 100101China

**Keywords:** Actin-binding protein, actin bundles, actin dynamics, *Arabidopsis thaliana*, fimbrin, pollen tube.

## Abstract

The actin cytoskeleton is increasingly recognized as a major regulator of pollen tube growth. Actin filaments have distinct distribution patterns and dynamic properties within different regions of the pollen tube. Apical actin filaments are highly dynamic and crucial for pollen tube growth. However, how apical actin filaments are generated and properly constructed remains an open question. Here we showed that Arabidopsis fimbrin5 (FIM5) decorates filamentous structures throughout the entire tube but is apically concentrated. Apical actin structures are disorganized to different degrees in the pollen tubes of *fim5* loss-of-function mutants. Further observations suggest that apical actin structures are not constructed properly because apical actin filaments cannot be maintained at the cortex of *fim5* pollen tubes. Actin filaments appeared to be more curved in *fim5* pollen tubes and this was confirmed by measurements showing that the convolutedness and the rate of change of convolutedness of actin filaments was significantly increased in *fim5* pollen tubes. This suggests that the rigidity of the actin filaments may be compromised in *fim5* pollen tubes. Further, the apical cell wall composition is altered, implying that tip-directed vesicle trafficking events are impaired in *fim5* pollen tubes. Thus, we found that FIM5 decorates apical actin filaments and regulates their organization in order to drive polarized pollen tube growth.

## Highlight

This study identifies FIM5 as an important player in constructing apical actin structure besides its defined role in constructing shank-oriented longitudinal actin cables in the pollen tube.

## Introduction

Pollen tube growth is an essential step during flowering plant reproduction (Lord and Russell, 2002; Berger *et al.*, 2008; McCormick, 2013) and a central question in plant reproductive biology is how the growth of the pollen tube is precisely regulated (Franklin-Tong, 1999; Hepler *et al.*, 2001). The regulatory role of the actin cytoskeleton during pollen tube growth has long been recognized and the specific roles of the key molecular players are becoming better understood. The actin cytoskeleton assumes distinct structures within different regions of the pollen tube (Lovy-Wheeler *et al.*, 2005; Ren and Xiang, 2007; Cheung and Wu, 2008; Chen *et al.*, 2009; Staiger *et al.*, 2010; Qin and Yang, 2011; Guan *et al.*, 2013; Qu *et al.*, 2015). It has generally been accepted that different actin structures carry out distinct functions within different regions of the pollen tube; for example, shank-oriented longitudinal actin cables are believed to provide molecular tracks to generate the reverse fountain cytoplasmic streaming pattern (Hepler *et al.*, 2001; Chebli *et al.*, 2013; Cai *et al.*, 2015). By comparison, the precise organization and function of apical actin structures are not well understood, although it is assumed that the apical actin structure is crucial for vesicle fusion and consequent apical cell wall patterning (Lee *et al.*, 2008; Kroeger *et al.*, 2009; Cheung *et al.*, 2010; Zhang *et al.*, 2010*b*; Bou Daher and Geitmann, 2011; Qin and Yang, 2011; Hepler *et al.*, 2013; Wang *et al.*, 2013; Rounds *et al.*, 2014). Decades of studies have allowed us to conclude that different actin structures are structurally, dynamically, and functionally distinct within different regions of the pollen tube. However, how pollen tubes generate and maintain distinct actin structures using the same building blocks within the common cytoplasm remains an open question.

There are at least five actin isovariants coexpressed in mature Arabidopsis pollen (Kandasamy *et al.*, 2002; Chang and Huang, 2015). This is different from the situation in yeast cells, which express only one actin isovariant (Novick and Botstein, 1985). Whether coexpression of different actin isovariants impacts the construction of distinct actin structures remains unclear. Construction of different actin structures requires the action of various actin-binding proteins (ABPs) (Staiger and Blanchoin, 2006; Higaki *et al.*, 2007; Thomas *et al.*, 2009; Huang *et al.*, 2015). Within the cytoplasm of a single cell, a given ABP may participate in the construction of different actin structures with distinct biochemical, biophysical, and dynamic properties. How this is achieved is an interesting question.

Fimbrin, also known as plastin, is a bona fide actin bundling protein that has been implicated in numerous fundamental, physiological cellular processes, including endocytosis, cytoplasmic streaming, and polarized cell growth (Wang *et al.*, 2010; Wu *et al.*, 2010; Jorde *et al.*, 2011; Skau *et al.*, 2011; Su *et al.*, 2012; Morley, 2013). There are five fimbrin-like genes encoded in the genome of *Arabidopsis thaliana* (Kovar *et al.*, 2000; Wu *et al.*, 2010). Among these, Arabidopsis fimbrin5 (FIM5) is preferentially expressed in pollen and has been implicated in the regulation of pollen germination and tube growth (Wu *et al.*, 2010). It was shown that FIM5 decorates actin filaments throughout the pollen tube, and its role in regulating the generation and maintenance of shank-oriented actin bundles has been well documented (Wu *et al.*, 2010). The lily (*Lilium longiflorum*) homologue of FIM5, Ll-FIM1, has been implicated in regulating the organization of actin structures within the subapical region of the pollen tube (Su *et al.*, 2012). However, neither the mechanisms by which FIM5 and Ll-FIM1 regulate the organization and dynamics of apical actin filaments nor the associated underlying cellular processes are well understood. Addressing this question promises to yield a better understanding of how exactly fimbrin regulates polarized pollen tube growth and will, in turn, enhance our understanding of the function of actin during pollen tube growth in general.

Here, we demonstrate that FIM5 is apically concentrated during pollen tube growth. Actin filaments become more curved in pollen tubes of *fim5* mutants, suggesting that the rigidity of actin filaments is compromised when *FIM5* function is lost. Consequently, apical actin filaments grow in different directions within the apical cytoplasm and cannot be maintained at the cortex. The dense cortical actin structure therefore fails to form in *fim5* pollen tubes. Unexpectedly, actin filaments become less dynamic in *fim5* pollen tubes. The apical actin filaments are more severely disorganized in some pollen tubes than in others which may explain why *fim5* pollen tubes exhibit different depolarization patterns as reported previously (Wu *et al.*, 2010). The alteration of apical cell wall composition allows us to speculate that FIM5-decorated apical actin structures are vital for tip-directed vesicle trafficking and secretion. Our study thus provides significant insights into the function and mechanism of action of FIM5, as well as shedding light on the function and mechanism of action of actin in general during pollen tube growth.

## Materials and methods

### Determination of the spatiotemporal subcellular distribution of FIM5 in pollen tubes

The subcellular distribution of FIM5 was examined by both laser scanning confocal microscopy and spinning disc confocal microscopy. Pollen tubes derived from *FIM5pro:FIM5-EGFP;fim5* (Wu *et al.*, 2010) were selected for observation under the microscope after their average length reached about 200 μm. To reveal more details of the subcellular distribution of FIM5, pollen tubes were observed under an Olympus FV1 000MPE multiphoton laser scanning confocal microscope equipped with a ×100 objective (N.A. 1.4). Projection images of longitudinal optical sections and transverse sections of FIM5-EGFP images were generated by ImageJ software (http://imagej.nih.gov/ij/). To enhance the temporal resolution of FIM5-EGFP, pollen tubes were visualized under a spinning disc confocal microscope and the z-stack time series images were collected at 2s intervals with the z-steps set at 0.5 μm. In order to quantify the fluorescent pixel intensity of FIM5-EGFP during pollen tube growth, a kymograph was drawn along the growth axis of the elongating pollen tube. Briefly, a line along the growth direction was initially drawn and the width was set to cover the whole pollen tube. Subsequently, the grey values along the line during the whole growth period were measured using an ImageJ plugin, StackprofileData (http://rsb.info.nih.gov/ij/macros/StackProfileData.txt) to generate a text file, which was then input into ImageJ to produce the kymograph. A threshold for fluorescence pixel intensity was set to distinguish the pollen tube region from the background after generating the kymograph and the fluorescence pixel intensity of the background was set to zero. Real-time average fluorescence pixel intensity within a region 5 μm distal to the tip was obtained and plotted over time. The rate of change of position of the first pixel with non-zero fluorescence intensity was counted as the velocity for the growing pollen tube and plotted over time.

### Visualization of the organization and dynamics of the actin cytoskeleton in pollen tubes

The organization of actin filaments was revealed by staining with Alexa-488 phalloidin in fixed pollen tubes as described previously (Wu *et al.*, 2010; Zhang *et al.*, 2010*a*). In order to visualize the dynamics of actin filaments in pollen tubes, they were decorated with Lifeact-EGFP as reported previously (Qu *et al.*, 2013). Images of actin filaments were captured by spinning disc confocal microscopy. Determination of the parameters associated with individual actin filaments was performed according to the published method (Zheng *et al.*, 2013). The physical properties of the actin filaments were evaluated by measuring the convolutedness, as well as the rate of change of convolutedness, as described previously (Staiger *et al.*, 2009; Qu *et al.*, 2013).

### Detection of cell wall components by immunostaining

Immunostaining of cell wall components was performed according to previously published methods (Yu *et al.*, 2009; Chebli *et al.*, 2012). Pollen tubes with an average length of about 200–300 μm were fixed in 4% paraformaldehyde in PEM (50mM PIPES, 5mM EGTA, 5mM MgCl_2_, pH 6.9) with 18% sucrose for 90min. They were subsequently washed with PEM once and PBS (137mM NaCl, 2.7mM KCl, 10mM Na_2_HPO_4_, 2mM KH_2_PO_4_, pH 7.0) twice. Primary antibodies were diluted in PBS with 3% BSA (bovine serum albumin) and incubated overnight at 4 °C followed by three washes in PBS with 3% BSA. Pectin with a low and high degree of esterification was detected with JIM5 and JIM7 antibodies (diluted at 1:100; Knox *et al.*, 1990), respectively. Callose was labelled with a monoclonal IgG antibody to (1→3)-β-glucan (Meikle *et al.*, 1991; diluted at 1:100). Crystalline cellulose was labelled with Cellulose Binding Module3a (CBM3a; diluted at 1:100; Blake *et al.*, 2006) followed by a monoclonal mouse anti-poly-His antibody (diluted at 1:800). Subsequently, samples were incubated with Alexa-488-conjugated donkey anti-rat IgG for JIM5 and JIM7 or Alexa-488-conjugated donkey anti-mouse IgG for (1→3)-β-glucan and CBM3a (diluted at 1:800 in PBS with 3% BSA) for 2h at room temperature, then washed three times with PBS containing 3% BSA. Pollen tubes were observed under an Olympus FV1 000MPE multiphoton laser scanning confocal microscope equipped with a ×100 objective (N.A. 1.4). The samples were excited under a 488-nm argon laser with the emission wavelength set at 505–605nm and fluorescent images were collected by Micromanager imaging software (Micro-Manager; https://www.micro-manager.org).

## Results

### FIM5 decorates filamentous structures throughout the pollen tube but is apically concentrated

**Fig. 1.**
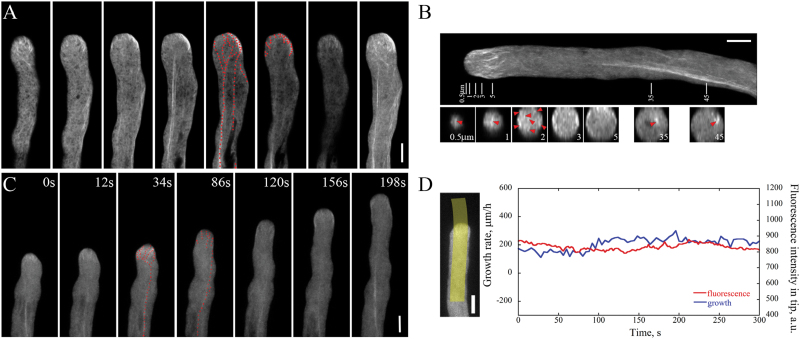
FIM5-EGFP decorates apical actin filaments. Subcellular localization of FIM5 in the pollen tube was revealed by visualizing *FIM5pro:FIM5-EGFP;fim5* under either a confocal laser scanning microscope as described previously (Wu *et al.*, 2010), or a spinning disc confocal microscope. (A) Z-series images of FIM5-EGFP in the pollen tube. The far right panel shows the projection image. Red dots indicate filamentous structures. Scale bar=5 μm. (B) Transverse sections showing FIM5-EGFP in the pollen tube. Red triangles indicate thick actin bundles. Numbers show the distance (in μm) of each transverse section from the apical tip of the pollen tube. Scale bar=5 μm. (C) Time-lapse images of FIM5-EGFP in the pollen tube. Scale bar=5 μm. The entire series is shown in Supplementary Movie S1. (D) Analysis of the average fluorescence pixel intensity of apical FIM5-EGFP and tube growth rates over time. The left panel indicates the region of interest used for the kymograph measurement. The right panel shows the graph plotted.

To decipher the precise function of FIM5, its spatiotemporal localization in the pollen tube was performed by visualizing *FIM5pro:FIM5-EGFP;fim5* pollen tubes as described previously (Wu *et al.*, 2010). To reveal more details of the spatial localization pattern of FIM5, optical sections of *FIM5pro:FIM5-EGFP;fim5* pollen tubes were collected under a confocal laser scanning microscope, allowing the generation of high spatial resolution projection images. Consistent with our previous observation (Wu *et al.*, 2010), we found that FIM5 is distributed throughout the pollen tube (Fig. 1A, B). Interestingly, we also noted that FIM5 concentrates at the pollen tube tip (Fig. 1A, B) which implies that FIM5 may play an even more prominent role in regulating apical filament organization than its defined role in organizing shank-oriented actin bundles (Wu *et al.*, 2010). To reveal the localization of FIM5 during pollen tube growth, we performed spinning disc confocal microscopy at high temporal resolution. Our results showed that the apically concentrated localization pattern of FIM5 is maintained during pollen tube growth (Fig. 1C, D; seeSupplementary Movie S1 at *JXB* online). This implies that apically concentrated FIM5 might be important for apical actin filament organization and polarized pollen tube growth.

### Apical actin filaments cannot form a dense cortical actin structure in *fim5* pollen tubes

**Fig. 2.**
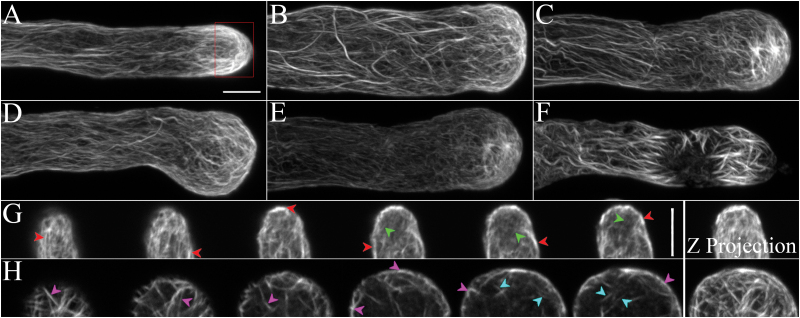
Actin filament structures become disorganized in *fim5* pollen tubes. Actin filaments in WT (Col-0) and *fim5* pollen tubes were revealed by staining with Alexa-488 phalloidin. (A) A typical WT pollen tube. The red box indicates the apical region of interest. Scale bar=5 μm for (A**–**F). (B–F) Several representative *fim5* pollen tubes showing different distribution patterns of actin filaments. (B, C) Pollen tubes with disorganized actin filaments throughout the entire tube and very prominent actin bundles in the shank region. (D) Pollen tube with actin filaments that show relatively normal distribution in the shank, but are disorganized in the subapical and apical regions. (E) Pollen tube with actin filaments that are thinner and disorganized throughout the tube (pollen tubes with this type of filament distribution were rarely detected). (F) Pollen tube with actin filaments that are disorganized throughout the entire tube, and severely disorganized in the subapical and apical regions. (G) Actin filament distribution in the apical region of a WT pollen tube. The images are z-series images and the far right panel is the z-projection image. Red arrowheads indicate dense actin filaments at the cortex and green arrowheads indicate actin filaments in the interior of the pollen tube. (H) Actin filament distribution in the apical region of a *fim5* pollen tube. The images are optical sections except for the far right panel which is a z-projection image. Purple and blue arrowheads indicate cortical and internal actin filaments, respectively. Scale bar=5 μm for (G and H).

To examine the function of FIM5 in regulating the organization of the apical actin structure, we used the null mutant *fim5-1* which had been characterized in our previous study (Wu *et al.*, 2010). We performed phalloidin staining for actin filaments in *fim5* pollen tubes and carefully compared the staining pattern with that in WT pollen tubes. Indeed, we found that actin filaments were abnormally organized throughout the entire pollen tube in *fim5* mutants and exhibited various patterns of disorganization (Fig. 2A–F; Wu *et al.*, 2010). In particular, the apical actin structure was disorganized to different degrees in *fim5* pollen tubes (Fig. 2A–F) and the dense apical actin structure found in WT pollen tubes (Fig. 2A; red boxed region) was not observed in *fim5* pollen tubes (Fig. 2B–F). To reveal more details of the defects in the apical actin structure, we examined z-series optical sections from the apical regions of WT and *fim5* pollen tubes (Fig. 2G, H). In WT pollen tubes, we found that most apical actin filaments are organized in a longitudinal direction, form relatively narrow angles with the pollen tube growth axis, and become concentrated at the cortex of the tube (Fig. 2G). By comparison, we found that apical actin filaments appeared to develop in mixed directions in *fim5* pollen tubes and did not concentrate at the cortex (Fig. 2H). The outcome of this may be that the dense cortical apical actin structure cannot form in *fim5* pollen tubes.

**Fig. 3.**
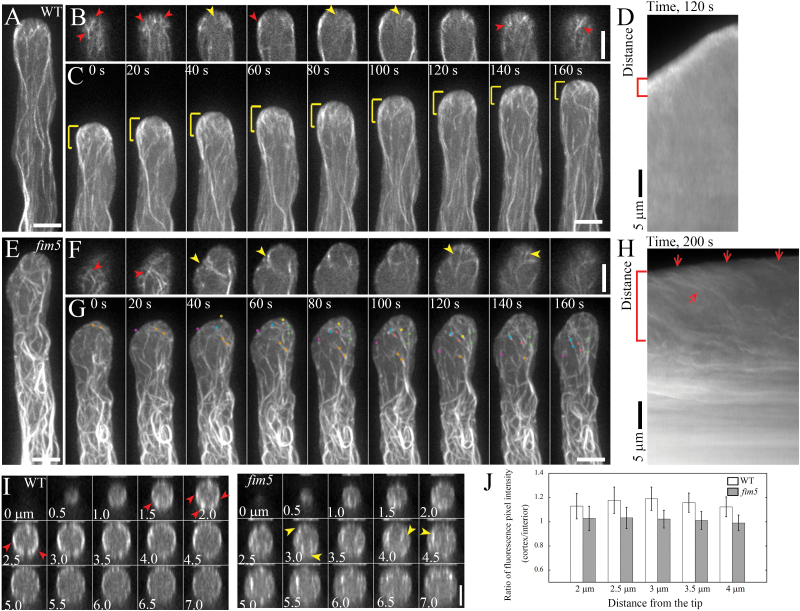
Apical actin filaments cannot be maintained at the cortex of *fim5* pollen tubes. (A–D) The regular bright actin structure (decorated with Lifeact-EGFP) generated at the apex of WT pollen tubes. (A) Maximum-intensity projection actin filament image of the individual z-stack in a WT pollen tube as shown in (B). Scale bar=5 μm. (B) Z-series optical sections of actin structures at the apex of the pollen tube shown in (A). Red and yellow arrows indicate cortical and interior actin filaments, respectively. Scale bar=5 μm. (C) Time-lapse projection images of actin filaments in a normally growing WT pollen tube. The dense apical actin structure is highlighted with yellow brackets. Scale bar=5 μm. (D) Kymograph of the growing pollen tube in (C). Apical actin is highlighted by the red bracket. Scale bar=5 μm. (E–H) Apical actin filaments (decorated with Lifeact-EGFP) became disorganized in a *fim5* pollen tube. (E) Maximum-intensity projection actin filament image of the individual z-stack in a *fim5* pollen tube as shown in (F). Scale bar=5 μm. (F) Z-series optical sections of actin structures at the apex of the pollen tube shown in (E). Red and yellow arrows indicate cortical and interior actin filaments, respectively. Scale bar=5 μm. (G) Time-lapse projection images of actin filaments in a *fim5* pollen tube. Several discriminatable filaments are indicated by different coloured dots. Scale bar=5 μm. (H) Kymograph of the growing pollen tube in (G). Apical actin is highlighted by the red bracket. Red arrows indicate actin filaments originating from the apical membrane. Scale bar=5 μm. (I) Transverse sections of actin filaments in a WT (left panel) and *fim5* pollen tube (right panel) are displayed. Dense cortical actin filaments in a WT pollen tube and thick actin filaments in a *fim5* pollen tube are highlighted by red and yellow arrows, respectively. Scale bar=4 μm. (J) Distribution of actin filaments in WT and *fim5* pollen tubes, quantified by dividing the fluorescence pixel intensity at the cortex of the tube by the intensity in the interior.

To reveal the details of actin filament dynamics, we performed live-cell imaging of actin filaments decorated with Lifeact-EGFP (Vidali *et al.*, 2009; Qu *et al.*, 2013). Consistent with the phalloidin staining results in fixed pollen tubes shown above, we found that apical actin filaments form relatively small angles with the tube growth axis in WT pollen tubes and concentrate at the cortex, consequently forming the dense cortical actin structure (Fig. 3A–C). Bright actin filaments are restricted to a small tip region in WT pollen tubes (Fig. 3D). By comparison, however, apical actin filaments are irregularly oriented in *fim5* pollen tubes and the dense cortical actin structure is not visible (Fig. 3E–G). Actin filaments originating from the apical membrane in *fim5* pollen tubes are dimmer and thicker than those in WT pollen tubes (Fig. 3H). These results suggest that the dense actin structure cannot form properly because the apical actin filaments are abnormally organized and cannot concentrate at the cortex of *fim5* pollen tubes. To support this conclusion further, we generated transverse sections for WT and *fim5* pollen tubes and found that actin filaments concentrate at the cortex in sections from the apical region of WT pollen tubes (Fig. 3I, left panel) but are distributed uniformly in the corresponding sections from the apical region of *fim5* pollen tubes (Fig. 3I, right panel). This was confirmed by quantitative measurement of the fluorescence pixel intensity of the transverse sections which showed that the ratio of the fluorescence pixel intensity of cortical actin filaments to that of interior actin filaments is lower in *fim5* pollen tubes compared with that in WT pollen tubes (Fig. 3J). Thus, these data suggest that dense apical actin structures cannot form properly in *fim5* pollen tubes because apical actin filaments cannot be maintained at the cortex.

### Apical actin filaments are less dynamic in *fim5* pollen tubes

**Fig. 4.**
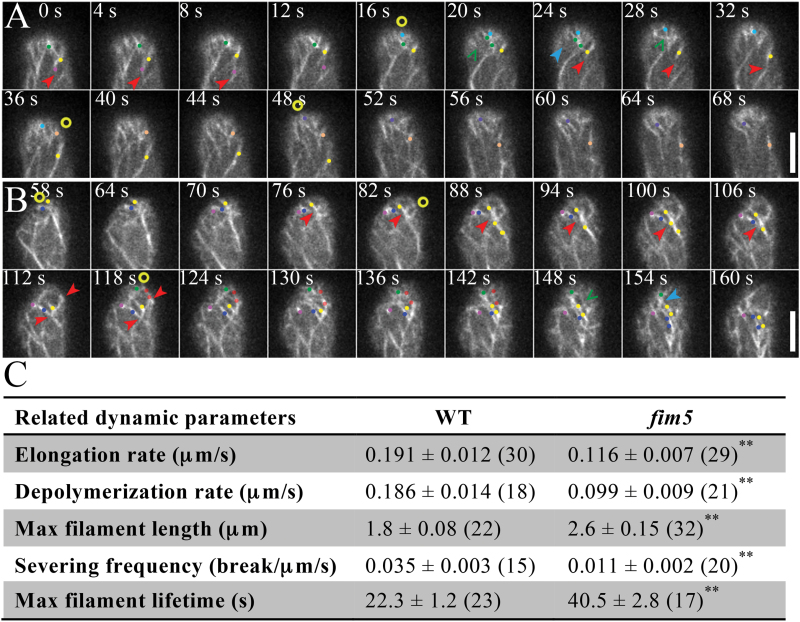
Apical actin filaments become less dynamic in *fim5* pollen tubes. (A, B) Time-lapse images of actin filament dynamics at the cortex of WT (A) and *fim5* (B) pollen tubes. The sites of origination of actin filaments are indicated by yellow circles. Actin filament elongation, depolymerization, and severing events are indicated by red, blue, and green arrows, respectively. Typical actin filaments are highlighted by different colored dots. Scale bar=5 μm in all images. (C) Dynamic parameters of actin filaments at the apical and subapical regions of WT and *fim5* pollen tubes. Measurements were taken from actin filaments in at least 10 pollen tubes. Values represent mean ±se, *P <0.05 and **P <0.01 by Student’s *t* test.

We next considered whether actin dynamics were altered in *fim5* pollen tubes. We therefore traced the dynamics of individual apical actin filaments and found that they underwent dynamic elongation, depolymerization, and severing events in WT and *fim5* pollen tubes (Fig. 4A, B). Consistent with the notion that apical actin filaments are less dynamic in *fim5* pollen tubes, we found that actin filament elongation and depolymerization rates and severing frequency were decreased in *fim5* pollen tubes compared with WT pollen tubes (Fig. 4C). This consequently leads to an increased actin filament lifetime in *fim5* pollen tubes (Fig. 4C). Thus, these data suggest that actin filaments become less dynamic in *fim5* pollen tubes.

### Actin filaments become more curved in *fim5* pollen tubes

**Fig. 5.**
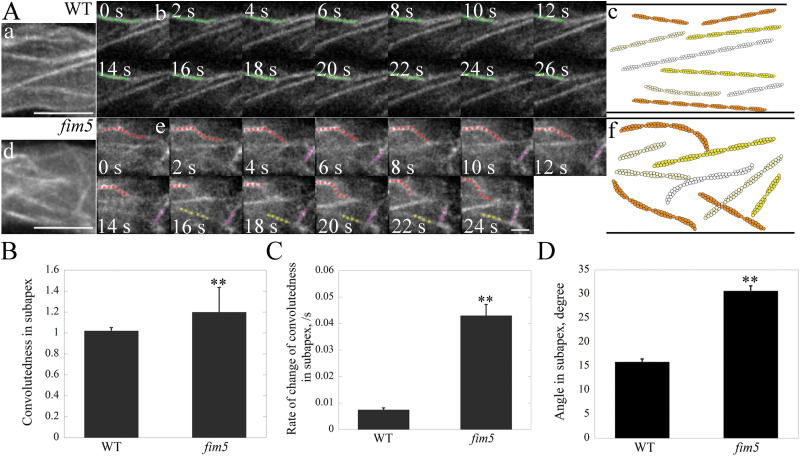
Actin filaments bend more easily at the subapex of *fim5* pollen tubes. (A) Time-lapse view of actin filaments at the subapex of WT and *fim5* pollen tubes. (a, b) Representative z-projection images and time-lapse optical sections of actin filaments at the subapex of a WT pollen tube. Scale bar=5 μm. An actin filament of interest is indicated by green dots. (c) Schematic distribution of actin filaments at the subapex of a WT pollen tube. (d, e) Representative z-projection images and time-lapse optical sections of actin filaments at the subapex of a *fim5* pollen tube. Scale bar=5 μm. Actin filaments of interest are indicated by red, purple, and yellow dots. (f) Schematic distribution of actin filaments at the subapex of *fim5* pollen tube. (B) The average convolutedness of actin filaments is significantly increased at the subapex of *fim5* pollen tubes. Data represent mean ±SE. **P <0.01 by Student’s *t* test. (C) The rate of change of convolutedness of actin filaments substantially increases at the subapex of *fim5* pollen tubes. Data represent mean ±SE. **P <0.01 by Student’s *t* test. (D) The average angles formed between actin filaments and the pollen tube growth axis increases at the subapex of *fim5* pollen tubes. Data represent mean ±SE. **P <0.01 by Student’s *t* test.

The decoration of actin filaments with certain actin bundling factors is reported to confer distinct biophysical and biochemical properties upon actin filaments (Klein *et al.*, 2004). We speculated that the biophysical and mechanical properties of actin filaments might be altered in *fim5* pollen tubes. In support of this hypothesis, we found that actin filaments became more curved in both the subapex and shank of *fim5* pollen tubes compared with WT pollen tubes (Fig. 5A; Supplementary Fig. S1A). To quantify the curvature of actin filaments in WT and *fim5* pollen tubes, we determined the convolutedness and the rate of change of convolutedness of actin filaments as described previously (Staiger *et al.*, 2009; Qu *et al.*, 2013). We found that both parameters were significantly increased in the subapex and shank of *fim5* pollen tubes compared with WT pollen tubes (Fig. 5B, C; Supplementary Fig. S1B, C). Consequently, the average angles formed between actin filaments and the tube growth axis are increased in the subapex and shank of *fim5* pollen tubes (Fig. 5D; Supplementary Fig. S1D). This explains why apical actin structures cannot form properly in *fim5* pollen tubes.

### Apical cell wall composition is altered in *fim5* pollen tubes

**Fig. 6.**
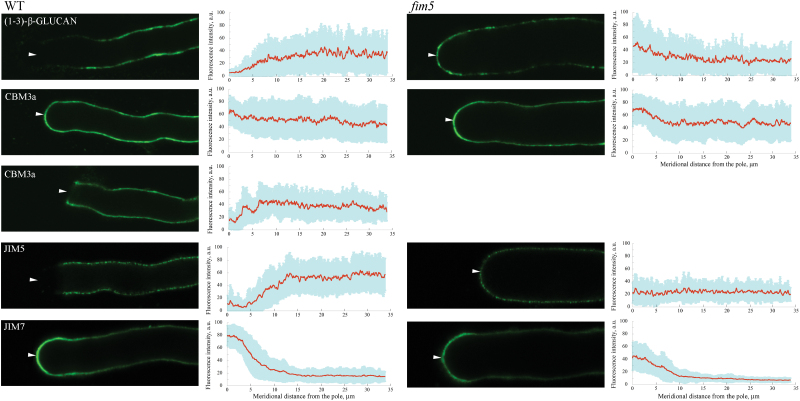
Loss of function of *FIM5* alters the cell wall composition at the pollen tube tip. Different components of the cell wall were revealed by immunostaining with antibodies against different cell wall antigens in WT and *fim5* pollen tubes. Callose was labelled with a monoclonal IgG antibody to (1→3)-β-glucan. Crystalline cellulose was labelled with CBM3a, followed by a monoclonal mouse anti-poly-His antibody. De-esterified pectin and esterified pectin were detected with JIM5 and JIM7 antibodies, respectively. The left panels are representative images of immunostained pollen tubes, and the right panels are the quantitative measurements of the fluorescence pixel intensity along the pollen tube from the tip. In each case, pixel intensities were measured in more than 30 immunostained pollen tubes. The red lines show the average fluorescence pixel intensity and the blue shaded regions indicate the SD.

The apical actin structure is believed to regulate secretion and retrieval of materials at pollen tube tips and, consequently, influences the structure of the tip, including the cell wall. We therefore wondered whether apical cell wall composition was altered in *fim5* pollen tubes. Callose and cellulose were present at low levels or absent from the apex of WT pollen tubes, but were concentrated at the apex of *fim5* pollen tubes (Fig. 6). Considering that the apical cell wall is mainly composed of a pectic network in normally growing pollen tubes (Ferguson *et al.*, 1998; Parre and Geitmann, 2005), we also performed staining for pectin with JIM5 and JIM7 antibodies to detect de-esterified and esterified pectin, respectively (Knox *et al.*, 1990). Pectin mainly exists in the esterified form and de-esterified pectin is hardly detectable at the apex of WT pollen tubes, whereas both pectin forms were detected at the apex of *fim5* pollen tubes (Fig. 6). The pectin and cellulose staining data suggest that the rigidity of the apical cell wall is increased in *fim5* pollen tubes, which would partially explain why *fim5* pollen tubes grow slowly. However, the amount of esterified pectin decreased in the shank of *fim5* pollen tubes (Fig. 6), suggesting that the incorporation of total pectin into the wall decreased in *fim5* pollen tubes. This actually suggests that secretion events are compromised in *fim5* pollen tubes. Taken together, these data indicate that the composition of the apical cell wall is altered in *fim5* pollen tubes, and that tip-directed vesicle trafficking events are impaired in *fim5* pollen tubes.

## Discussion

**Fig. 7.**
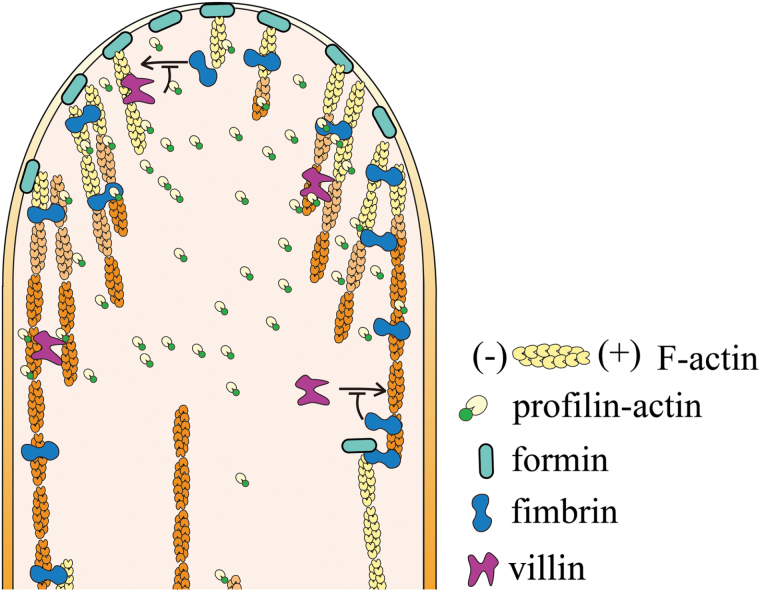
Schematic diagram describing the function of FIM5 in regulating the organization of apical actin filaments. Based on previous results (Cheung *et al.*, 2010; Liu *et al.*, 2015), we assumed that apical actin filaments are nucleated by formins from the apical membrane utilizing profilin–actin complexes. Based on the localization pattern of FIM5 and the effect of loss of function of FIM5 on apical actin filament organization, we hypothesized that FIM5 decorates apical actin filaments when they are nucleated which allows them to maintain a certain level of bundling and to grow in regular directions towards the apical cytoplasm. Consequently, this process leads to the generation of dense cortical actin structures within the apical and subapical regions of the pollen tube. FIM5 co-ordinates with other actin bundling factors (villin in particular) to ensure the proper behaviour of apical actin filaments.

Here we demonstrate that FIM5 plays a very prominent role in regulating the construction of apical actin structures in the pollen tube. FIM5 decorates actin filaments throughout the entire pollen tube but is apically concentrated and this apically-concentrated localization pattern is maintained during pollen tube growth, suggesting that FIM5-decoration is very likely required for the construction of apical actin structures in the pollen tube. Consistent with this hypothesis, apical actin filaments became disorganized in *fim5* pollen tubes, probably because the rigidity of the actin filaments is compromised, as evidenced by the increase in convolutedness of the filaments in *fim5* mutants. Therefore, we propose that FIM5 decorates apical actin filaments to regulate their bundling and to confer physical and mechanical properties upon them which ensures appropriate growth behaviour from the apical membrane to allow the construction of dense apical actin structures in the pollen tube (Fig. 7). Our study provides significant insights into the molecular mechanism underlying the construction of the apical actin structure.

Within pollen tubes, actin filaments are assembled into distinct actin structures which are assumed to perform different functions (Cheung and Wu, 2008; Staiger *et al.*, 2010; Qin and Yang, 2011; Guan *et al.*, 2013; Hepler and Winship, 2015; Qu *et al.*, 2015). How actin filaments are assembled into different structures within the common cytoplasm in pollen tubes remains largely unclear. Although different actin structures are structurally and morphologically distinct within different regions of the pollen tube, they are actually spatially connected (Qu *et al.*, 2015). This is different from the situation in other systems: in yeast cells for instance, actin filaments are assembled into three major structurally distinct and spatially separated actin structures, actin rings, actin patches, and actin cables (Kovar *et al.*, 2011). Given that assembly of distinct actin structures is controlled by the co-ordinated action of various ABPs, it is very interesting to determine how a certain ABP regulates the construction and maintenance of these spatially connected distinct actin structures within the common cytoplasm of the pollen tube. As a bona fide actin bundling protein, fimbrin is expected to play important roles in regulating the construction of different actin structures in the pollen tube. Indeed, a previous study identified FIM5 as being important for regulating the construction of longitudinal actin cables in the shank which, in turn, are essential for cytoplasmic streaming (Wu *et al.*, 2010). However, the role of FIM5 in the construction of apical actin structures has been overlooked, although its homologue, Ll-FIM1, has been implicated in regulating the organization of actin filaments in the subapical region of the pollen tube (Su *et al.*, 2012). Furthermore, the underlying mechanism by which fimbrin regulates the organization and dynamics, as well as the function of apical actin filaments remains to be characterized. In this study, inspired by the finding that FIM5 is apically concentrated during pollen tube growth (Fig. 1), we performed the careful characterization of the function and mechanism of action of FIM5 in regulating the construction of apical actin structures.

Consistent with the apically concentrated localization pattern, we found that apical actin filaments exhibit different degrees of disorganization in *fim5* pollen tubes (Figs 2, 3). This is very likely due to the fact that apical actin filaments are not regularly organized in *fim5* pollen tubes, unlike in WT pollen tubes (Fig. 2G, H). This was further verified by the live-cell imaging of apical actin filaments which showed that they grow out from the apical membrane in mixed directions (Fig. 3). The disorganization of apical actin structures is probably due to the decrease in the rigidity of actin filaments, as evidenced by the decrease in convolutedness of actin filaments in both subapical and shank regions of *fim5* pollen tubes (Fig. 5; Supplementary Fig. S1). These results suggest that FIM5 decoration is required to maintain the appropriate biophysical properties of actin filaments, thus ensuring that they grow in a way that allows the construction of dense and regular apical actin structures in the pollen tube. It is reasonable to expect that loss of FIM5 alters the biophysical properties of actin filaments, but we did not predict that actin filaments would become less dynamic in *fim5* pollen tubes (Fig. 4). We do not currently know why loss of an actin bundling factor down-regulates actin dynamics, but we speculate that it may alter the composition of ABPs on actin filaments that, in turn, down-regulates the action of several factors that promote actin turnover, such as actin depolymerizing factor (ADF) and its cofactors (Smertenko *et al.*, 2001; Allwood *et al.*, 2002; Chen *et al.*, 2002; Ketelaar *et al.*, 2004; Chaudhry *et al.*, 2007; Deeks *et al.*, 2007; Augustine *et al.*, 2011; Shi *et al.*, 2013; Zheng *et al.*, 2013). Future genetic analyses together with *in vitro* reconstitution experiments may provide clues as to whether the decrease in actin dynamics in *fim5* pollen tubes results from an alteration in the composition of ABPs.

In terms of the alteration of ABP composition, we predict that the most direct consequence will be the up-regulated binding of other actin bundling factors resulting from the loss of FIM5. We did not detect a decrease in the bundling level of actin filaments in *fim5* pollen tubes (Fig. 2; Wu *et al.*, 2010), which indeed implies that the binding of other actin bundling factors is up-regulated in *fim5* pollen tubes. One of the candidate bundling factors will be Arabidopsis villins, which were shown to be bona fide actin filament bundlers (Bao *et al.*, 2012; Zhang *et al.*, 2010*a*) and are required for the proper organization of actin filaments (Qu *et al.*, 2013). Although it was proposed that villin-mediated actin filament severing activity is vital for the proper organization of apical actin filaments (Qu *et al.*, 2013), their function in regulating the organization of longitudinal actin bundles in the shank of pollen tubes is mainly contributed by their actin filament bundling activity, where the concentration of calcium is low. In fact, the contribution of the bundling activity of villins to the proper organization of apical actin filaments needs to be considered and examined. In this aspect, the disorganization of actin filaments in *fim5* pollen tubes may, to some extent, result from the disrupted balance between FIM5 and villins on actin filament bundling. In support of this notion, previous *in vitro* reconstitution experiments demonstrate the formation of mixed fimbrin–villin–actin bundles (Glenney *et al.*, 1981). More work need to be undertaken to verify this. Certainly, other bundling factors, like LIM domain containing proteins (LIMs) (Papuga *et al.*, 2010), should also be taken into account in the future. This may consequently lead to differential interaction between actin filaments and actin turnover-promoting factors. In support of this hypothesis, it was demonstrated that FIM1 co-operates with tropomyosin conditionally to regulate the action of cofilin in fission yeast (Skau and Kovar, 2010). The decrease in actin filament dynamics also explains why more long apical actin filaments appear in the apical region of *fim5* pollen tubes compared with WT pollen tubes (Fig. 3D, H).

The next interesting question is why and how FIM5 concentrates on apical actin filaments within the common cytoplasm of pollen tubes. Considering that at least five reproductive actin isovariants are expressed in mature pollen (Kandasamy *et al.*, 2002; Chang and Huang, 2015), it is possible that different actin structures have different actin isovariant compositions within distinct regions of the pollen tube. This may result in differential interaction between actin filaments and FIM5. In addition, it has been proposed that the initial binding of some ABPs strengthens the recruitment of a specific set of ABPs during the construction of different actin structures. For instance, it has been proposed that the nature of actin structures is determined when actin assembly is first initiated by actin nucleation factors. These nucleation factors subsequently recruit specific sets of ABPs to allow the formation of distinct actin structures. This leads to the notion that the generation of a certain actin structure is determined when it was born (Michelot and Drubin, 2011). Therefore, it is possible that actin structures within different regions have a specific ABP composition that leads to the differential recruitment of FIM5 in the pollen tube. Considering that the formins have been demonstrated or postulated to be major actin nucleation factors in the pollen tube (Ye *et al.*, 2009; Cheung *et al.*, 2010; Liu *et al.*, 2015), it is possible that actin filaments nucleated by different formin isovariants may allow differential recruitment of various ABPs within the common cytoplasm of the pollen tube. Finally, it is well known that the cytoplasm in the pollen tube contains different zones, each having distinct properties, e.g. different concentrations of ions such as [Ca^2+^] and [H^+^] (Cheung and Wu, 2008). Therefore, some ABPs may exhibit differential actin filament binding activity within different regions of the pollen tube. Indeed, some Ca^2+^- and H^+^-responsive F-actin binding ABPs have been reported in plants including gelsolin-like domain-containing proteins (Huang *et al.*, 2004; Xiang *et al.*, 2007; Khurana *et al.*, 2010; Zhang *et al.*, 2010*a*, 2011), ADF (Carlier *et al.*, 1997; Smertenko *et al.*, 2001; Chen *et al.*, 2002; Bou Daher *et al.*, 2011 ), and LIMs (Papuga *et al.*, 2010; Gui *et al.*, 2014). In the case of FIM5, the affinity of its binding to actin filaments might be higher within the apical region compared with other regions in the pollen tube. It will take further work to explore the possibilities proposed above.

The fact that apical actin filaments display diverse patterns of disorganization in *fim5* pollen tubes (Fig. 2) may explain why *fim5* pollen tubes exhibit different depolarization patterns as reported previously (Wu *et al.*, 2010). Although we cannot link specific patterns of disorganization to specific patterns of depolarization, we propose that the variable levels of disorganization of apical actin filaments differentially affect tip-directed vesicle trafficking and accumulation, as well as the secretion events at the pollen tube tip. In support of this, we found that the composition of apical cell wall components is altered in *fim5* pollen tubes (Fig. 6) since the the composition of the cell wall is subjected to regulation by vesicle trafficking. Determining the link between apical actin filament disorganization and pollen tube depolarization in *fim5* mutants will enhance our understanding of the mechanism of action and function of apical actin structures in the pollen tube. Our previous findings showed that the velocity of cytoplasmic streaming decreases in *fim5* pollen tubes. To some extent, this explains why *fim5* pollen tubes grow slowly, but not why they exhibit different depolarization patterns. In view of this point, the present study complements our previous work (Wu *et al.*, 2010) and enhances our understanding of the function and mechanism of action of FIM5, as well as the actin cytoskeleton in general in the pollen tube.

## Supplementary data

Supplementary data can be found at *JXB* online.

Figure S1. Actin filaments bend more easily in the shank of *fim5* pollen tubes.

Movie S1. Dynamic localization of FIM5-EGFP in a growing pollen tube.

Supplementary Data
