# Supplementary material for: Arabidopsis FIM5 decorates apical actin filaments and regulates their organization in the pollen tube
Source: J Exp Bot. 2016 Apr 25;67(11):3407–17. doi: 10.1093/jxb/erw160 (PMC4892729; doi:10.1093/jxb/erw160)
Supplement: Supplementary Data [file supp_67_11_3407__index.html]

Arabidopsis FIM5 decorates apical actin filaments and regulates their organization in the pollen tube — Arabidopsis FIM5 decorates apical actin filaments and regulates their organization in the pollen tube — Supplementary Data 

# Arabidopsis FIM5 decorates apical actin filaments and regulates their organization in the pollen tube

## Supplementary Data

Data files

- Supplemental\_Figure\_S1.pdf - Supplementary Data
- Supplemental\_Movie\_S1.avi - Supplementary Data
