# Supplementary figures and images for: Arabidopsis FIM5 decorates apical actin filaments and regulates their organization in the pollen tube
Source: J Exp Bot. 2016 Apr 25;67(11):3407–17. doi: 10.1093/jxb/erw160 (PMC4892729; doi:10.1093/jxb/erw160)

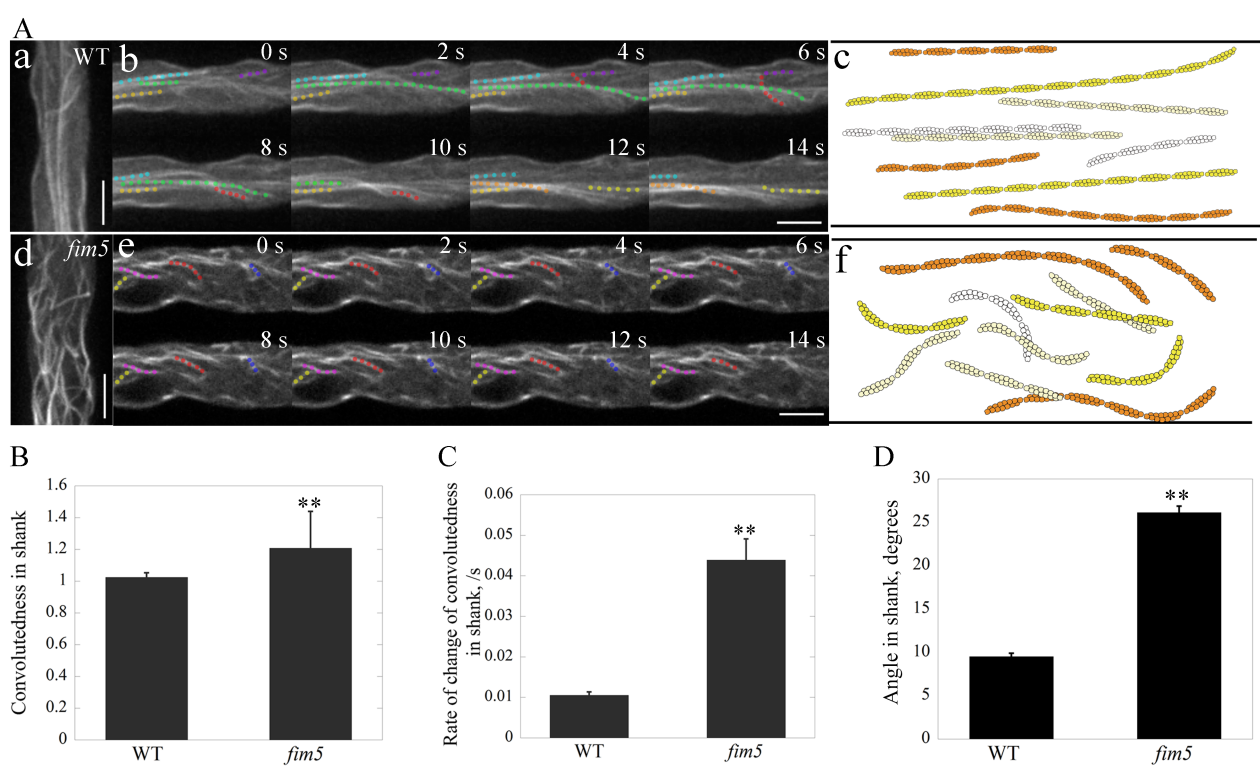

Figure S1

Supplement: Supplementary Data [file supp_erw160_Supplemental_Figure_S1.pdf]
